# Development of a Flexible Strain Sensor Based on PEDOT:PSS for Thin Film Structures

**DOI:** 10.3390/s17061337

**Published:** 2017-06-09

**Authors:** Alexandra El Zein, Camille Huppé, Cédric Cochrane

**Affiliations:** 1 ENSAIT, GEMTEX, F-59100 Roubaix, France; alexandra.elzein@gmail.com (A.E.Z.); camille.huppe@alumni-ensait.fr (C.H.); 2 University Lille Nord de France, F-59000 Lille, France

**Keywords:** flexible piezoresistive sensor, PEDOT:PSS, intrinsically conductive polymer, strain gauge

## Abstract

The aim of this study was to develop and optimize a reproducible flexible sensor adapted to thin low-density polyethylene (LDPE) films and/or structures to enable their deformation measurements. As these deformations are suspected to be weak (less than 10%), the developed sensor needs to be particularly sensitive. Moreover, it is of prime importance that sensor integration and usability do not modify the mechanical behavior of its LDPE substrate. The literature review allowed several materials to be investigated and an elastomer/intrinsically conductive polymer PEDOT:PSS (Clevios^TM^) filled composite was selected to simultaneously combine mechanical properties and electrical conductivity. This composite (made of PEDOT:PSS and silicone Bluesil^®^) presented satisfying compatibilities with piezoresistive effects, negative temperature performances (in a range from −60 °C to 20 °C), as well as elongation properties (until the elastic limit of the substrate was reached). The method used for creating the sensor is fully described, as are the optimization of the sensor manufacture in terms of used materials, the used amount of materials where the percolation theory aspects must be considered, the adhesion to the substrate, and the manufacturing protocol. Electromechanical characterization was performed to assess the gauge factor (*K*) of the sensor on its substrate.

## 1. Introduction

To maintain competitiveness, one way to sustain technical innovations is to reinforce scientific and technological foundations and increase the performance of existing structures. To meet this challenge, an in-depth knowledge of structure mechanisms is required. For this purpose, sensors are exceedingly interesting as they enable interactions with our physical world by transforming a biological, chemical, or physical phenomenon such as strain deformation, into a measurable electrical variable [1]. However, when the key issue is to measure the weak deformations of a thin and highly flexible host structure, conventional gauges (made of gold, silver, copper, platinum) are unsuitable as their mechanical behavior cannot compete with flexible substrates [2]. These are limited to micro-strain measurements, whereas flexible conductive polymer sensors can still perform at larger tensile strains [3]. Conductive polymers are comprised of conductive polymer composites (CPC), which are obtained by blending an insulating polymer matrix with conductive fillers, and intrinsically conductive or semi-conductive polymers (ICP) [2]. Compared to metal, conductive polymers based strain gauges, either made of CPCs (carbon or metallic particles) or intrinsically conducting polymers (ICPs) are interesting not only for their lower cost, outstanding flexibility, or light weight [4,5,6]; but also for their simple processing as either melt-mixed solid compounds (carbon or metallic particles), or as liquid dispersions or solutions for film-forming and coating [2,7]. Moreover, they advantageously share the electrical properties of metals and the mechanical properties and process ability of common polymers [5]. Thus, they fulfill the mechanical requirements of the substrates while being utilized as active strain gauge layers. ICPs are inherently highly conductive owing to the presence of conjugated double bonds in their molecular structure [2], thus giving rise to conjugated π-electrons [1,7]. Their solubility is improved by doping, especially for polyaniline (PANI) or polythiophenes such as poly(3,4-ethylenedioxythiophene)-poly(styrene sulfonate) (PEDOT:PSS). For instance, PEDOT ensures polymer conductivity while PSS (through an insulator) connects PEDOT to water [3]. Thus, water-based PEDOT:PSS dispersions of high quality can be achieved [1]. PEDOT:PSS is often considered as one of the most successful ICPs [2,7]. For example, it is easier to process than polyaniline (PANI) [8] and possesses an increased radiation-transparent property compared to carbon black. Compared to other conductive materials under standard atmospheric conditions, it has an increased stability and degradation resistance and seems to be less sensitive to oxidation [1]. However, film forming from commercially formulated PEDOT:PSS is of little interest as it exhibits brittle behavior and is prone to cracking [9]. This may be explained by the fact that commercial formulations containing PEDOT:PSS were developed to suit optical and conductive properties; therefore, the development of a flexible active layer requires blending PEDOT:PSS with an elastomer (thermoplastic or not) such as latex or silicone, which demonstrate excellent film-forming abilities [2,9]. The elastomer acts as a matrix, which provides improved mechanical and adhesion properties to the blend, while PEDOT:PSS ensures electrical conductivity [2,9]. Moreover, PEDOT:PSS (as do all conductive polymers) exhibits a piezoresistive property [3]: its electrical resistance depends on the elongation applied to the material [1,2,7], which justifies the use of conductive polymers as strain gauges [7]. This dependence is characterized by the gauge factor (*K*). Table 1 synthesizes the gauge factor of several materials. This electrical dependence is either due to a change in sensor geometry (for instance, a decreased cross-section of the sensor causes an increased resistance of the conductive material), or an intrinsic modification of the sensor (for instance, a modified interaction between the polymer and fillers particles), or the superimposition of both effects (an increased temperature implies thermal expansion of the polymer, resulting simultaneously in a geometrical change and a modified inherent conduction mode) [1,2,10]. The filler content within the elastomer/conductive polymer blend also influences its electrical properties [2]. However, other parameters such as adhesion properties on the substrate, mechanical behavior, or aesthetic finishing could be less favorable than expected. Thus, a compromise between the piezoresistive response and other global characteristics of the material is often necessary for the sensor, while maintaining a low resistance and satisfying the electrical signal [2]. A wide range of applications require the use of piezoresistive sensors: in the medical field as well as in sport, they enable body monitoring either for rehabilitation and surveillance of clinical signs, or for improving performances [7,11,12]. Other fields of interest can be found in monitoring structures such as airbags, wind sailing, or turbines; structural composites; or parachute canopies [2,7]. In this paper, the development and characterization of a flexible piezoresistive sensor is described. This study echoes our laboratory’s expertise in textiles and previous studies on piezoresistive sensors [2,6,7,9]: as textiles present a unique combination of flexibility, elasticity, etc., and are lightweight, they easily deform under minimal stresses [2], presenting a mechanical behavior like that of thin films. Moreover, textiles, being the closest barriers to the skin, are able to follow all human gestures, thus making them ideal candidates for the incorporation of smart sensors [8,11,12,13,14,15], including piezoresistive sensors. Furthermore, the coating techniques for textiles are similar in principle to the ones used for polymer depositions on different substrates.

This paper aimed to obtain a flexible, reproducible and lightweight sensor to record the deformations of a specific structure, while respecting simple and rapid prototyping processes. Moreover, the substrate deformations were suspected to be weak (less than 10%). First, the selected polymers and the method used to develop the strain gauge piezoresistive sensor to provide electrical data over a wide range of temperatures, are fully described. In particular, the sensor was designed as a water-based thermoplastic elastomer (silicone)/intrinsically conductive polymer (PEDOT:PSS) composite. Next, sensor characterization was performed in terms of conductive, mechanical and electromechanical properties. As the developed sensor is deposited by direct coating on thin and flexible substrates, it is therefore of prime importance that the embedded sensor does not alter their mechanical behavior. The piezoresistive properties of the coating were calibrated to obtain satisfactory adhesion properties, as well as acceptable sensor sensitivity. There, the reproducibility of the sensor performances—a point of high interest—was characterized through electromechanical tests, where the change in resistance was recorded based on the applied stress. Finally, the obtained results are discussed.

## 2. Materials and Methods

### 2.1. Materials

#### 2.1.1. Sensor Substrate

The sensor substrate was a thin multilayer film made of low-density polyethylene (LDPE) provided by our industrial partner, from whom the project originates.

**Figure 1:**
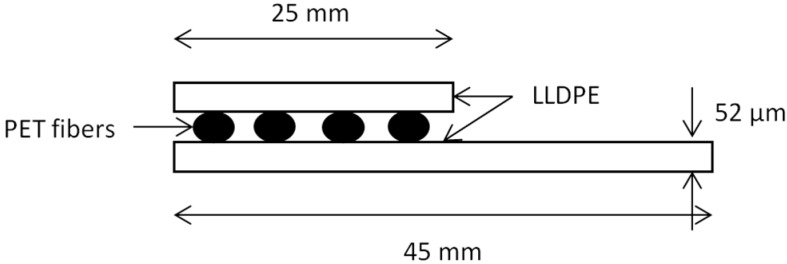
Description of the sensor substrate.

The substrate was 46 mm wide and included polyethylene terephthalate (PET) fibers between its layers (shown in Figure 1). The PET fibers were covered by another LDPE film that was 25 mm wide. Each film was 52 μm thin. For the experimental tests, the samples dimensions were 300 mm × 45 mm.

#### 2.1.2. Conductive Filler

A wide range of commercial dispersions of PEDOT:PSS has been developed to meet end-users expectations. A commercial formulation of PEDOT:PSS (Clevios^TM^ P form 105D) was provided by the Heraeus Company, Leverkusen, Germany. Typically, this dispersion is developed for anti-static coatings as it has good adhesion properties on plastics, especially on polyethylene films and glass substrates [21]. The dispersion contained 1.2% of solid content [21], and the composition is presented in Table 2 [21].

#### 2.1.3. Matrix

For flexible mechanical sensor applications, a conductive filler could be used with a polymer matrix to improve mechanical behavior and adhesion with the substrate. The electrical conductive blend between the conductive filler and insulating matrix is generally called the conductive polymer composite (CPC). In this study, an aqueous acrylic latex solution (Appretan^®^ 96100 from Clariant, Paris, France) was used. As it has a glass transition temperature (Tg) around −20 °C, the mechanical properties of the latex at low temperatures (beyond −20 °C) were weak and the coating made of a PEDOT:PSS/latex blend separated from the tested substrates due to poor adhesion. Consequently, the latex matrix was discarded in favor of a two-component water-based silicone emulsion (Bluesil^®^ TCS 7110 A&B from Bluestar Silicone, Lyon, France, solid content 54% for Part A, 40% for Part B, which is a catalyst).

#### 2.1.4. Protection of the Sensor and Connecting Wires

To prevent sensor degradation under inappropriate manipulation or friction, a sealant silicone layer (available in large and common do-it-yourself stores) was deposited on the dried coating made of PEDOT:PSS and Bluesil^®^ silicone.

The electrical signal provided by the sensor was transmitted to a computer to be recorded and analyzed. This transmission was performed by flexible stainless steel wires (from Bekintex, Wetteren, Belgium). The 505 Tex wires are composed of 2 × 275 filaments having an individual diameter of 12 μm.

### 2.2. Preparation of the Sensor

The conductive composites sensors made of PEDOT:PSS blended with silicone were fabricated in five steps:

1. Preparation of aqueous dispersions (silicone and PEDOT:PSS);
2. Preparation of the substrate;
3. Deposition of the conductive layer;
4. Deposition of the connecting wires; and
5. Deposition of a protective silicone layer.

#### 2.2.1. Preparation of Aqueous Dispersions (Silicone and PEDOT:PSS)

To produce the conductive polymer composites (CPC) solution, 87.5 mL of PEDOT:PSS dispersion was stirred on a magnetic stir plate for 5 min, then heated and stirred around 90 °C until it lost 76.7% of liquid content and was finally removed from heat. Next, 10 mL of the silicone Part A was added and stirred for 5 min. Finally, 1 mL of the silicone Part B was added and stirred for 5 min. Different rates of PEDOT:PSS/aqueous silicone were tested and deposited on the substrate to determine the percolation threshold.

#### 2.2.2. Preparation of the Substrate

As LDPE is hydrophobic, surface activation on both sides of each substrate was preliminarily realized by plasma treatment. The experimental parameters were set at 500 W and a speed of 4 m/min. This well-known treatment results in good hydrophilic surfaces with an increased ability for interaction-forming [5]; thus, the PEDOT:PSS/silicone blend had better adhesion on the treated substrates. Moreover, the Clevios Coating Guide [21] strongly recommends performing a plasma treatment prior to depositing the coating.

#### 2.2.3. Deposition of the Conductive Layer

Once the CPC solution cooled down, it was deposited onto the plasma-treated substrates using a syringe. The substrates were first recovered by a piece of 100 mm × 10 mm × 20 μm rectangular, thin, stainless steel. The blend deposited at one end of the track was then wet-coated on the other end using a glass blade. The mask was then removed and the samples were dried at room temperature for 4 h. For each batch of CPC solution produced, two sets of at least six coatings were obtained to verify experimental reproducibility.

The drying phase was of prime importance. During the sample drying, PEDOT:PSS (as well as silicone particles) compacted due to solvent evaporation [8,22]. According to References [8,22], water evaporation conducts the formation of a segregated microstructure where the free PSS chains are forced to form the outer insulating layer of the coating. Moreover, within the coating, PEDOT:PSS particles cannot deform enough to eliminate all void spaces [22], and are therefore carried to the coating surface due to internal pressure, low density and water-swell [22]. The presence of remaining water could be a serious issue when considering sensor performance: as the developed sensor is deposited on a structure subjected to high variations of temperature (from −60 to 40 °C) as well as relative humidity, the trapped water inside the coating could freeze, leading to signal interference, or a loss in coating adhesion to the structure.

The length/width ratio impacts on sensor electrical performance (geometrical effect), according to Equation (1) is

(1) R=ρ×L/S

where R (Ω) is the electrical resistance of the material, ρ (Ω·m) is the specific resistivity; L its length (m); and S its cross-sectional area (m^2^). During the coating phase, a 100 mm long and 10 mm wide PEDOT:PSS/Silicone track was deposited on the LDPE substrates. Despite a rather weak length/width ratio, the chosen sensor dimensions fit with the final applications for which the sensor was developed and was enough to discriminate the transverse (vertical) piezoelectric effect of the sensor during electromechanical tests.

#### 2.2.4. Deposition of the Connecting Wires

To transmit the electrical signal provided by the sensor, flexible stainless steel wires were connected at both extremities of the sensor over a length of around 20 cm. The extremities of the wires (in contact) with the conductive track, were opened widely to collect the substrate deformations on the total width of the track. Next, these areas were covered with a small drop of the previous PEDOT:PSS/Silicone blend to ensure good electrical connection upon drying. There, the fineness of the filaments was of prime importance since the total width of the track had to be covered in the most homogeneous manner possible. Finally, the uncoated extremities were connected to isolated copper wires.

#### 2.2.5. Deposition of a Protective Silicone Layer

Finally, to protect the whole sensor system, a protective sealant silicone layer was deposited on the dried conductive coating. This deposit may have many advantages:

- As a chemically inert and waterproof material, it may protect the conductive coating from the harsh outside environment (especially from UV radiation);
- It may reinforce the adhesion properties of the coating on the substrates;
- Due to the overall dark blue color of the coating, the sensor may heat when exposed to direct sunlight (because of IR absorption), leading to signal interferences (because of exceeding dilatation), or advanced ageing. As the chosen sealant silicone was white, it also may lower the sun’s influence on sensor performance.

To check the influence of silicone on the mechanical behavior of the sensors, the layer was 130 mm long for varying tested widths, ranging from 20 to 40 mm. These dimensions are given in Table 3. The application of the sealant silicone layer was confined by a removable mask, using the same method as the conductive coating deposition. The obtained silicone film was approximately 50 μm thick on Substrate 1 (measured with an optical profilometer Cotec Altisurf 500 on five samples). A surface treatment was added to sample SP-40 to prevent coating delamination from very low temperatures.

**Figure 2:**
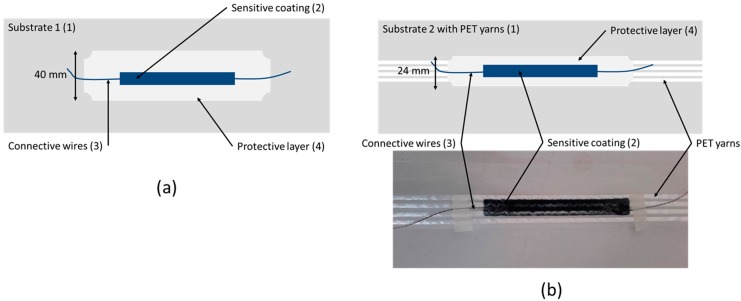
Sensor scheme and real sensor on low-density polyethylene (LDPE) film (**a**) without and (**b**) with polyethylene terephthalate (PET) yarns.

The structure of the final sensor system appears in Figure 2. There, the sensors were prepared using a transparent sealant silicone to see the conductive coating. However, the protective layer developed for industrial purposes is white (IR shielding).

As the substrates are particularly thin and flexible, they deform easily. Thus, a serious issue was to ensure that the conductive coating and the protective layer were deposited without affecting the mechanical behavior of the substrates. This was of prime importance since this study aimed to create a sensor for very weak deformations. Therefore, the substrates were mechanically tested prior and post depositions of the conductive coating and protective layer made of sealant silicone.

Eight samples were tested, three of which were coated with the conductive blend, while the others remained uncoated.

For characterization of the protective layer, four replicas of each sample type were prepared. Table 3 identifies these tested samples.

The final sensor (Figure 2b) was electromechanically tested, as was the junction of the connecting wires.

### 2.3. Test Methods for the Characterization of Prepared Sensor

#### 2.3.1. Electrical Resistance of the PEDOT:PSS/Silicone Coatings

To characterize coating conductivity and consequently optimize the sensors based on the specific needs of the study, the best mass ratio between PEDOT:PSS and silicone must be found to ensure the greatest possible sensitivity and reproducibility to the sensors. Consequently, several sensors with varying weight ratios of PEDOT:PSS and silicone were prepared as previously described. However, the protective layer, being useless for such a test, was not deposited on these samples.

●  Testing equipment

**Figure 3:**
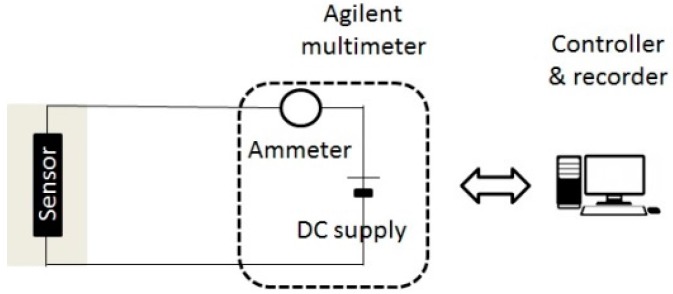
Experimental set-up for the coating conductivity measurement.

The coating conductivity measurements were performed using an Agilent multimeter by connecting alligator clips to the stainless-steel wires. The clips were put at 2 cm from the extremities of the coating. The experimental set-up is shown in Figure 3 [2].

●  Expression of results

Voltage was applied to the sensor, which varied from −0.5 to 10 V, with an automatic increment of 0.1 V. The current intensity I was measured, and the I/V curve was plotted for each set of sensors. Hence, electrical resistance *R* (Ω) was deduced from the slope of the linear part of the curve. The electrical conductivity of the sensor was established from the plotted log (R) curve as a function of varying weight ratios of PEDOT:PSS.

Four replicas of each equal ratio were prepared and tested.

#### 2.3.2. Mechanical Characterization

●  Validation of the global flexibility

Mechanical tests ensured that the protective layer deposition was intimately deposited on the substrate surface and could follow its deformations without affecting its original mechanical behavior. Thus, the flexibility of the original substrate was preserved, hence validating the design of the sensor system.

Mechanical characterization was investigated using a universal tensile strength tester (MTS 2/M) bench where jaw speed was programmed, load was recorded and the axial component of strain was controlled.

The standard parameters of the test procedure employed were (ISO 13934-1: 2013):

- Initial distance of the jaws: 200 mm
- Speed of the jaws: 100 mm/min
- Preload: 0 N

The protective layer deposited on the substrate was positioned parallel to the direction of the extension, and at the center of the tested sample.

The samples were subjected to uniaxial stretching under standard atmosphere (20 °C and 65% relative humidity).

Mechanical characterization was obtained by plotting the applied stress as a function of the relative elongation $\varepsilon_{r}$ (the strain) of the samples, which is defined by Equation (2).

(2) $$\varepsilon_{r}=\left(L-L_{0}\right)/L_{0}$$

where *L* (mm) is the extended length of the sample; and *L*_0_ is the initial length between the jaws (*L*_0_ = 200 mm).

●  Validation of the yarn connection

This tensile test determines the minimal force at break applied to the yarn of the sensor when it breaks from the external layer.

Mechanical characterization was investigated using a universal tensile strength tester (MTS 2/M) bench where jaw speed was programmed, load was recorded and the axial component of strain was controlled.

The standard parameters of the test procedure employed were (ISO 13934-1: 2013):

- Initial distance of the jaws: 200 mm
- Speed of the jaws: 100 mm/min
- Preload: 0 N

**Figure 4:**
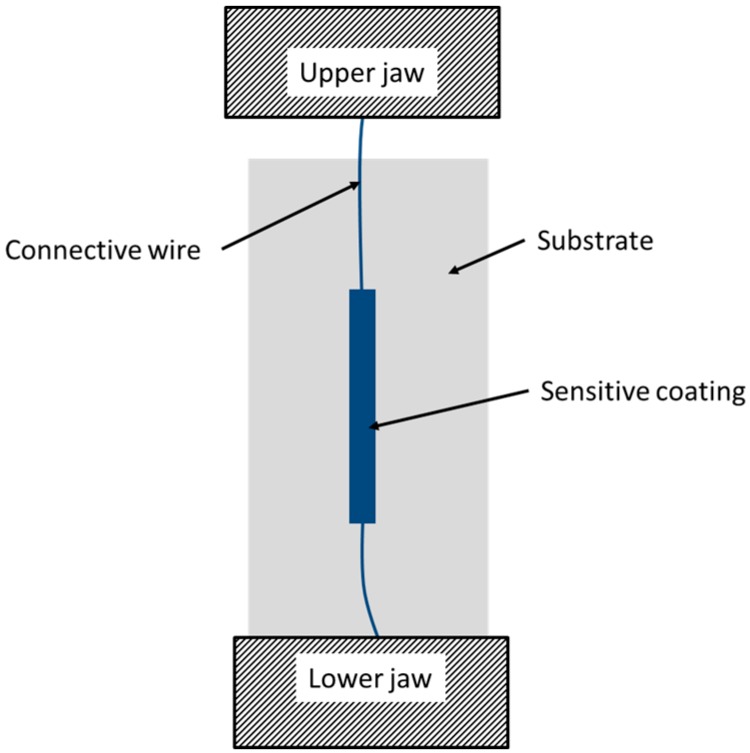
Tensile test bench.

The sample was caught in the lower jaw on one side. On the other side, the yarn was caught in the upper jaw as seen in Figure 4. The protective layer deposited on the substrate was positioned parallel to the direction of the extension, and at the center of the tested sample.

The samples were subjected to uniaxial stretching under standard atmosphere (20 °C and 65% relative humidity). Two results were obtained for each sample.

#### 2.3.3. Electromechanical Characterization of the Sensor

The reproducibility and the performance as a weak strain gauge of the sensor systems were studied through electromechanical tests. Furthermore, the influence of the plasma treatment and the protective layer deposited on the gauge factor were characterized.

**Figure 5:**
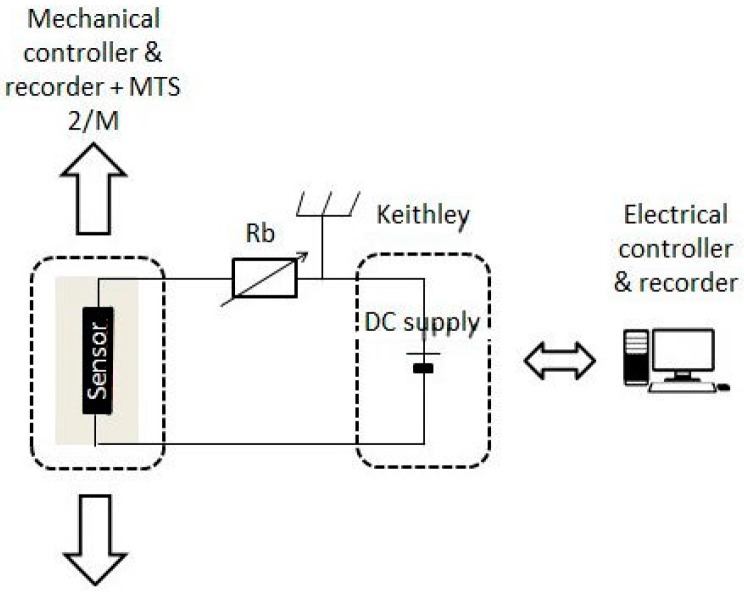
Experimental set-up for electromechanical tests.

The electrical response of the sensor to strain was recorded over time by a data acquisition system with a built-in voltage source from Keithley. The internal power supply provided a voltage set at 5 V and allowed for measurements with high accuracy. This apparatus was connected to a computer to record, plot, and process the sensor electrical voltage during the tensile elongation of the substrate. A simple divider bridge was implanted between the Keithley and the MTS 2/M bench, which recorded the strain applied to the sensor over its deformation. The bridge resistance *R_b_* was adjusted to obtain an initial voltage of 2.5 V. The experimental set-up is shown in Figure 5.

The sensor electrical resistance was obtained using the simple divider bridge, as defined in Equation (3).

(3) $$R=\left(V_{s}\times R_{b}\right)/\left(V_{k}-V_{s}\right)$$

where $V_{s}\;\left(V\right)$ is the measured sensor voltage$;\;V_{k}\;\left(V\right)$ is the voltage of the power supply provided by the Keithley apparatus; $R_{b}$ (Ω) is the bridge resistance; and R (Ω) is the electrical resistance of the sensor during the measurements.

A test procedure similar to that described in Section 2.3.2 was used for recording the mechanical behavior of the sensors. The sensor was caught in the jaws. The conductive track was positioned parallel to the direction of the extension, and at the center of the tested sample. During the tests, given the disposition of the connecting wires on the conductive track, the current flowed in the same direction as the sensor extension. Thus, the longitudinal piezoresistive effect was measured. Six sensors systems were prepared as described in the methods.

The recorded signal from the sensor was plotted over the strain applied, the obtained elongation and the time spent. Data were expressed and a normalized relative resistance was defined by (Equation (4)) to characterize the electrical response of the sensors independently from the resistance variations observed from an individual sensor to another.

(4) $$R_{n}=\left(R-R_{0}\right)/R_{0}$$

where R (Ω) is the electrical resistance of the sensor during the measurements; and $R_{0}\;\left(\Omega \right)$ its initial value (without extension).

The results were obtained by plotting the variation of the normalized relative resistance $R_{n}$ versus the sensor relative elongation $\varepsilon_{r}$. The slope of the obtained curve was the gauge factor *K*, as defined in Equation (5) as follows:

(5) $$K=R_{n}/\varepsilon_{r}.$$

## 3. Results and Discussion

### 3.1. Conductivity of the PEDOT:PSS/Silicone Coatings

**Figure 6:**
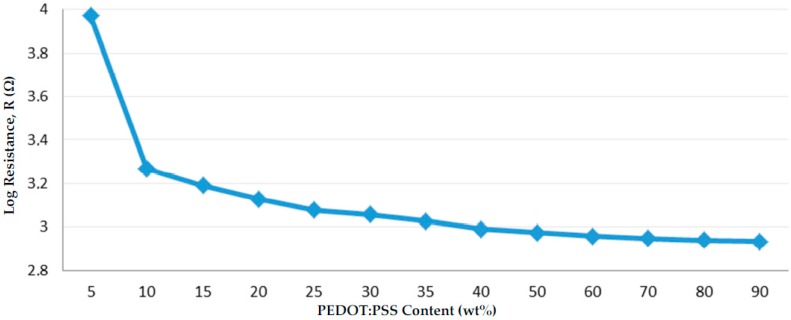
Conduction behavior of the sensor in a percolation-like theory.

Resulting from the linear I/V curves, the conduction behavior of the coatings for varying weight ratios of PEDOT:PSS to Silicone is shown in Figure 6.

From 5 to 10 wt % of dried PEDOT:PSS, the curve indicated the steepest slope, although the resistance did not vary with much magnitude. Subsequently, the percolation threshold, which is usually identified by a dramatic drop in resistance, was suspected to happen between 2 and 5 wt % of the PEDOT:PSS. At this critical concentration, the sensor sensitivity reached a climax due to numerous conductive areas of PEDOT:PSS that were close enough to form electro-conductive channels [8]. Consequently, the prior electrically insulated material became conductive.

However, when considering the use of the piezoresistive effect, the plotting method was the most adapted. The obtained percolation threshold was in the same range compared to the one comprised between 1 and 3 wt % PEDOT:PSS, achieved by Åkerfeldt et al. [8] working on a blend mostly composed of PEDOT:PSS (unique filler) and polyurethane. Moreover, our result was consistent with some of the percolation thresholds achieved by using other intrinsically conductive polymer solutions made of conductive filler, especially PANI, and an insulating polymer used as a matrix material [22,23].

Nevertheless, the obtained blend below 5 wt % was not the easiest to coat due to low-viscosity. Good coating behavior was obtained for two formulations, containing PEDOT:PSS/Silicone in proportions of 10/90 and 15/85, respectively. On one hand, both formulations partially benefitted from the percolation, even if the prepared sensor had a lesser sensitivity. On the other hand, they were low enough—considering inner electrical resistance—to avoid noise generation in the perceived signal. Consequently, a good compromise between sensor sensitivity and resistance value was obtained. Both formulations were selected for further experiments. When subjected to electromechanical tests at negative temperatures, the 15/85 PEDOT:PSS/Silicone formulation was preferred due to better adhesion properties at −60 °C as the silicone part was more important.

### 3.2. Response of the Sensors to Mechanical Strain

#### 3.2.1. Validation of the Global Flexibility

**Figure 7:**
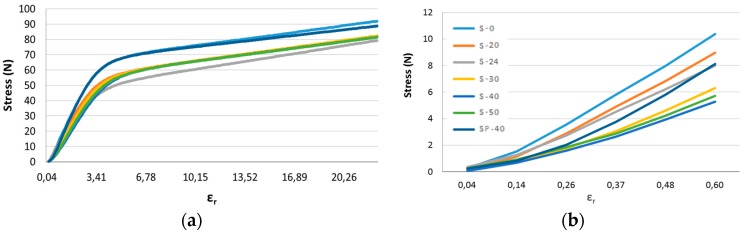
Mechanical behaviors of protected or unprotected samples: (**a**) overview; and (**b**) closer view.

Figure 7 enabled us to determine whether the conductive coating deposit affected the initial mechanical behavior of the substrate. We observed that all profiles were similar, and differences in behavior may have been due to the slightly different climatic conditions of the test, or due to the operator. Consequently, the conductive coating deposit did not appear to affect the original mechanical behavior of the substrate. Figure 7 also allowed us to determine the mechanical influence of the protective sealant-silicone layer on the substrate, and the tested samples are listed in Table 3. Compared to the sample used as a reference (S-0), the other samples presented a similar profile, and we observed that the added protective layer tended to slightly rigidify the substrate.

The protective layer has no influence on sensor’s mechanical behavior as well as plasma treated substrate. Electromechanical tests are performed with plasma treatment and a 25 mm width.

#### 3.2.2. Validation of the Yarn Connection

Table 4 shows the results of the tensile strength test. Two connecting wire junctions were tested by the sensors. The minimum force at break was about 15 N. In real applications and with the correct use of the device, the connection wire would never be pulled. Next, the minimum force of 15 N was sufficiently high to validate the mechanical strength of the sensor junction.

### 3.3. Electromechanical Characterization of the Sensors

**Figure 8:**
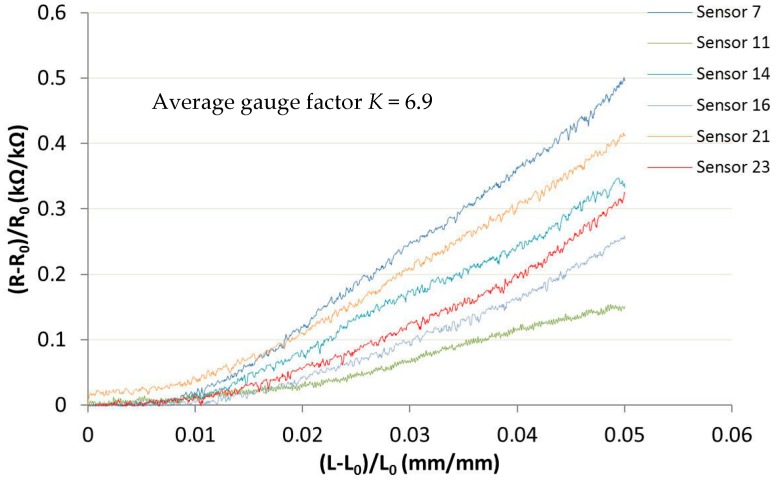
Electromechanical results of the sensors.

The electrical responses of some of the sensors composed of 15/85 PEDOT:PSS/Silicone are shown in Figure 8. Six out of a batch of 30 sensors were randomly selected and used for the electromechanical test. The others were used for other types of characterization.

When the atmospheric balloon took off, the elongation was very small (about 3%), which was why the electromechanical test was carried out with a 5% elongation.

The average gauge factor of the linear equations for each curve is given in Figure 8 (*K* = 6.9). The gauge factor is represented, but had a low value and the curves were dispersed. Moreover, it was not representative as it did not fit very well with the curves, which was why Equation (6) was used to fit with the curves.

(6) $$Rn=a\varepsilon_{r}^{p}$$

**Figure 9:**
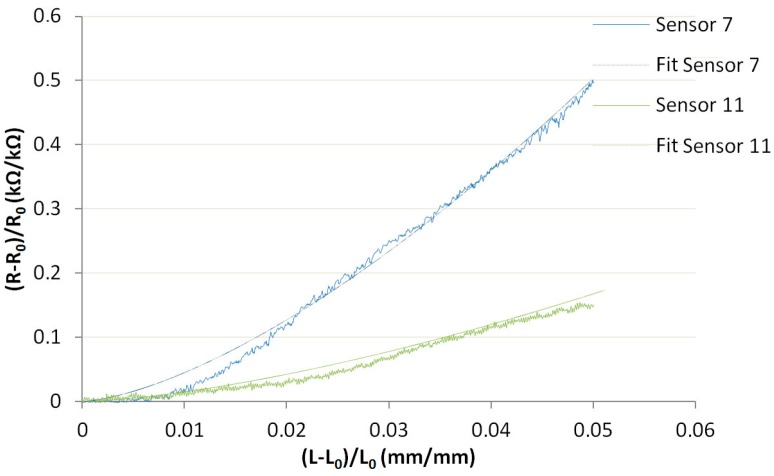
Electromechanical results of two sensors with fit curves.

In Equation (6), *p* is the constant and worth 1.5 [24], and *a* is similar to the gauge factor. As presented in Figure 9, this model was more representative of the date and value as *a* ranged from 15 (Sensor 11) to 45 (Sensor 7), and the average of the 6 samples was 28.83.

The obtained average gauge factor can be increased by achieving a PEDOT:PSS/Silicone ratio closer to the percolation threshold; however, a similar study focused on thin films showed that the increased gauge factor only ranged from 1 to 3 [7]. Furthermore, previous tests undertaken in this study clearly showed that the 15/85 ratio demonstrated the best abilities concerning substrate adhesion at negative or room temperatures, coating integrity during mechanical tests, or coating viscosity.

The sensor response resulted from both the geometrical effect and a change in the percolation network system due to the reorganization of their internal structure during elongation, or their competition [7,8]. Any given material with a finite elastic modulus will deform when subjected to applied stress; yet, its electrical resistance depends on the given geometry of the material (defined in Equation (1)) [1]: subjected to longitudinal extension, the cross-sectional area of the sensor shrinks while length increases, resulting in increased resistance.

As length increases, there may be a reorganization of the electrical paths or connections due to the molecular arrangement of the coating. Within the coating, the PEDOT:PSS forms a network of polymer chains; the coating surface is PSS rich, while the rest of the coating is more likely to be PEDOT rich [22]. When subjected to elongation, the PEDOT and PSS rich areas may align themselves according to the stress direction [3]. Accordingly, the conductive PEDOT areas may be in an unstable position and tend to ensure a more stable one, thus leading to a change in the electrical resistance of the sensor. Likewise, other molecular reorganizations might occur at the PEDOT:PSS/protective sealant-silicone layer, PEDOT:PSS/aqueous silicone, or PEDOT:PSS/substrate interfaces [2].

The coating implementation process on films is complicated, and different methods have been tested to reduce dispersion, but it is still significant. A big part of this research is to improve this process to reduce the sensors’ dispersion.

## 4. Conclusions

The development of a flexible and lightweight strain gauge sensor based on a conductive polymer composite, where the conductive part was ensured by the intrinsically conductive polymer PEDOT:PSS, was undertaken. This sensor was designed to measure particularly low deformations, whilst being subjected to temperatures ranging from −60 to 20 °C. To address these challenges, the sensor was optimized in terms of dimensions, geometry, design, preparation protocol, ease of processing, and filler concentration. The composition of the coating formulation, despite it not being optimal in terms of conductivity properties, is of prime importance in obtaining both satisfactory conductivity, and good adhesive properties to the substrate. Mechanical characterization showed that the created sensor system respected the original mechanical behavior of the substrate, and electromechanical testing was used to further validate the system. The obtained mechanical and electromechanical results provide an encouraging step forward in the further development of elongation sensors and their potential for broader applications as a conductive polymer for flexible technologies. Future studies will look at optimizing sensor reliability and repeatability, and to perform further validation.

## Figures and Tables

**Table 1:** Gauge factors and applications of several materials.

Materials	Gauge Factor (*K*)	Application
Gold [1]	2.1	Traditional strain gauge
Copper [1]	2.2	Traditional strain gauge
Platinum [1]	4.0	Traditional strain gauge
Constantan or Karma [16]	2.1	Alloys for metallic strain gauge
Nichrome V [16]	2.5	Alloy for metallic strain gauge
Isoelastic [16]	3.5	Alloy for metallic strain gauge
Platinum-Tungsten [16]	4.1	Alloy for metallic strain gauge
Silver [17]	3.35	Ink used for strain sensor applied by aerosol jet
Commercial semi-conductors [16]	45–175	Strain gauge
PEDOT:PSS [17]	0.48	Ink used for strain sensor applied by coating
PEDOT:PSS [18]	17.8 ± 4	Electrochemical synthesis of a PEDOT:PSS thin film deposited on a textile substrate
PMMA-MWCNT Composite [17]	15.32	Ink used for strain sensor made by hot pressing
PMMA-MWCNT Composite [1,19]	235	Micro pressure sensor chip
Carbon black-Evoprene [2]	80	Strain gauge on textile substrate
ESL/Silver + graphite pastes [20]	≈5	Thick film strain sensors using screen-printing technique

**Table 2:** Composition of the PEDOT:PSS Clevios^TM^ P form 105D by Heraeus.

Component	% By Weight
Clevios^TM^ F010	42.92
*N*-Methyl-2-pyrrolidinone	2.58
Sliquest^®^ A 187^TM^	0.86
Isopropanol	53.34
Dynol^TM^ 604	0.30
Total	100.00

**Table 3:** Tested samples for mechanical characterization of the protective layer deposit.

Sample Number	Description of the Sample	Dimension of the Protective Layer (Length × Width)	Total Number of Tested Samples
S-0	Substrate 1 used as reference	Without protective layer	4
S-20	Substrate 1 + protective layer	130 mm × 20 mm	4
S-24	Substrate 1 + protective layer	130 mm × 24 mm	4
S-30	Substrate 1 + protective layer	130 mm × 30 mm	4
S-40	Substrate 1 + protective layer	130 mm × 40 mm	4
S-50	Substrate 1 + protective layer	130 mm × 50 mm	4
SP-40	Plasma treated substrate 1 + protective layer	130 mm × 40 mm	4

**Table 4:** Tensile strength test results.

Force at Break Average (N)	Force at Break Minimum (N)	Standard Deviation	CV %	Samples Number
19.00	15.13	2.83	15	9
